# mir-124-5p Regulates Phagocytosis of Human Macrophages by Targeting the Actin Cytoskeleton via the ARP2/3 Complex

**DOI:** 10.3389/fimmu.2019.02210

**Published:** 2019-10-04

**Authors:** Estefania Herdoiza Padilla, Peter Crauwels, Tim Bergner, Nicole Wiederspohn, Sabrina Förstner, Rebecca Rinas, Anna Ruf, Michael Kleemann, René Handrick, Jan Tuckermann, Kerstin Otte, Paul Walther, Christian U. Riedel

**Affiliations:** ^1^Institute of Microbiology and Biotechnology, University of Ulm, Ulm, Germany; ^2^Central Facility for Electron Microscopy, Ulm University, Ulm, Germany; ^3^Institute of Applied Biotechnology, University of Applied Sciences Biberach, Biberach, Germany; ^4^Institute of Comparative Molecular Endocrinology, Ulm University, Ulm, Germany

**Keywords:** microRNA, macrophage, phagocytosis, high-content screening, ARP2/3

## Abstract

Phagocytosis is a cellular process crucial for recognition and removal of apoptotic cells and foreign particles, subsequently initiating appropriate immune responses. The process of phagocytosis is highly complex and involves major rearrangements of the cytoskeleton. Due to its complexity and importance for tissue homoeostasis and immune responses, it is tightly regulated. Over the last decade, microRNAs (miRNAs) have emerged as important regulators of biological pathways including the immune response by fine-tuning expression of gene regulatory networks. In order to identify miRNAs implicated in the regulation of phagocytosis, a systematic screening of all currently known, human miRNAs was performed using THP-1 macrophage-like cells and serum-opsonized latex beads. Of the total of 2,566 miRNAs analyzed, several led to significant changes in phagocytosis. Among these, we validated miR-124-5p as a novel regulator of phagocytosis. Transfection with miR-124-5p mimics reduced the number of phagocytic cells as well as the phagocytic activity of phorbol-12-myristate-13-acetate (PMA)-activated THP-1 cells and *ex vivo* differentiated primary human macrophages. *In silico* analysis suggested that miR-124-5p targets genes involved in regulation of the actin cytoskeleton. Transcriptional analyses revealed that expression of genes encoding for several subunits of the ARP2/3 complex, a crucial regulator of actin polymerization, is reduced upon transfection of cells with miR-124-5p. Further *in silico* analyses identified potential binding motifs for miR-124-5p in the mRNAs of these genes. Luciferase reporter assays using these binding motifs indicate that at least two of the genes (*ARPC3* and *ARPC4*) are direct targets of miR-124-5p. Moreover, ARPC3 and ARPC4 protein levels were significantly reduced following miR-124-5p transfection. Collectively, the presented results suggest that miR-124-5p regulates phagocytosis in human macrophages by directly targeting expression of components of the ARP2/3 complex.

## Introduction

Phagocytosis is a complex immunobiological process by which extracellular particles are actively internalized into membrane-surrounded vesicles termed phagosomes by professional phagocytes such as macrophages (MΦ) or dendritic cells (1). As part of the innate immune system, this process is essential for host defense against invading microorganisms (1–3). Furthermore, phagocytic clearance of senescent or damaged cells by MΦ is crucial to maintain tissue homeostasis (4). The phagocytic process is initiated by specific binding of surface receptors on macrophages to their respective ligands on the target (1, 5). Upon binding, receptor-specific downstream signaling cascades trigger cytoskeleton remodeling by partial disassembly of cortical actin and simultaneous recruitment and activation of actin nucleation proteins (5). Subsequently, pseudopods are formed toward phagocytic targets that facilitate engulfment. This is mediated by actin filament branching via the actin-related protein 2/3 (ARP2/3) complex leading to formation of the phagocytic cup (5–7). Due to its complexity, phagocytosis is a strictly regulated process.

In recent years, miRNAs have emerged as powerful regulators of gene expression and function in eukaryotes (8, 9). MiRNAs are small (18–22 nucleotides), endogenous, non-coding RNA molecules that regulate the expression of different target mRNAs in a sequence-specific manner (8). They are transcribed by RNA-polymerase II and further cleaved for maturation by the RNase-III enzymes DROSHA and DICER. Mature miRNAs are incorporated into the RNA-induced silencing complex by binding to proteins of the Argonaute family. The incorporated strand serves as a guide that specifically recognizes binding sequences in target mRNAs. Depending on the degree of complementarity to target mRNA transcripts, miRNA binding can promote Argonaute-catalyzed mRNA decay (high complementarity) or transcriptional repression (low complementarity).

Using transcriptional profiling, a number of miRNAs were identified that are differentially expressed during development, differentiation, and activation of human and murine macrophages and following encounter of microbial antigens (10–15). As up to 90% of the mammalian genes are regulated by miRNAs, miRNAs are also expected to modulate the process of phagocytosis in MΦ (16). However, a systematic, genome-wide investigation of miRNA-dependent regulation of phagocytosis is missing.

In recent years, high-content miRNA screens have been used as a successful strategy to identify miRNAs that regulate a number of cellular pathways including apoptosis and necrosis (17), hedgehog signaling (18), smooth muscle cell proliferation, and vascularization (19). Furthermore, gain-of-function high-throughput screenings have allowed identification of miRNAs involved in cancer (20–22), cardiopathology (23, 24), as well as their characterization as biomarker candidates (25). Here, we utilize a comprehensive high-content screening of miRNA in human MΦ to discover novel miRNAs involved in phagocytic clearance. We validate one candidate in detail, identify target mRNAs, and demonstrate the regulation of ARPC3, and ARPC4 two crucial components of the ARP2/3 complex.

## Results

### Screening System to Identify miRNAs Regulating Phagocytosis

**Figure 1:**
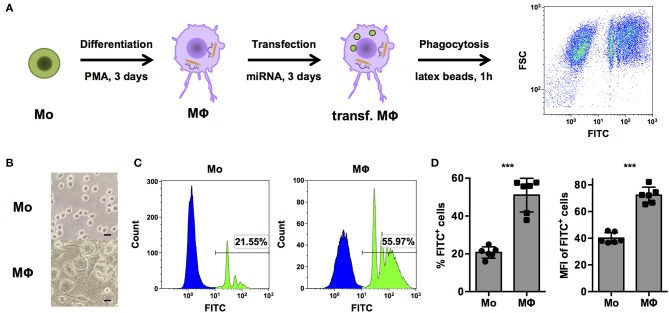
PMA-activated THP-1 cells allow investigation of phagocytosis of opsonized latex beads by human MΦ. **(A)** Schematic representation of the workflow for differentiation, transfection, and analysis of phagocytosis of FITC-labeled, opsonized latex beads for high-content screening of a miRNA library and subsequent experiments. **(B**–**D)** Bright-field microscopy and FACS analysis of untreated (Mo) or PMA-activated (MΦ) THP-1 cells. **(B)** Microscopic images were acquired with an AxioObserver Z1 microscope (Zeiss) using a 40× objective. Scale bars represent 20 μm. **(C)** Histogram plots of one representative experiment and **(D)** quantification of the percentage of FITC^+^ (left) and mean fluorescence intensity (MFI) of FITC^+^ cells (right) of *n* = 6 independent experiments. In **(D)**, values from each experiment are indicated by symbols and the mean ± standard deviation (SD) are indicated by a bar and whiskers. Statistical analysis was performed using Student's *t*-test (***p < 0.001).

In order to systematically identify miRNAs that are able to regulate phagocytosis in human macrophages, a cell-based assay to screen a library comprising mimics of all currently known, human miRNAs was established in THP-1 cells (Figure 1A). THP-1 cells are widely model cell line for human monocytes (Mo) and MΦ and were differentiated from a monocytic phenotype into macrophage-like cells by stimulation with phorbol 12-myristate 13-acetate (PMA). As observed in previous studies (26), PMA treatment induced morphological changes from small, round monocyte-like cells to considerably enlarged, more granular THP-1 MΦ with cellular protrusions (Figure 1B). Propidium iodide (PI) staining was used to exclude necrotic cells. PMA activation slightly reduced viability, i.e., the PI-negative (PI^−^) population of THP-1 MΦ compared to unstimulated THP-1 Mo (70.5 ± 30.3% vs. 96.4 ± 7.90%; *p* = 0.07, *n* = 6, data not shown). More importantly, the percentage of PI^−^ FITC^+^ THP-1 MΦ containing latex beads was approximately 2.5-fold higher than for Mo (51.1 ± 8.9% vs. 20.7 ± 3.0%; *p* < 0.001; Figures 1C,D). Enhanced phagocytosis was observed not only by an increased proportion of PI^−^ FITC^+^ cells but also in the phagocytic activity on the single-cell level. The mean fluorescence intensity (MFI), which correlates to the amount of internalized beads, of the PI^−^ FITC^+^ population was increased from 40.1 ± 4.1 in Mo to 72.3 ± 6.0 in MΦ (*p* < 0.001; Figure 1D).

**Figure 2:**
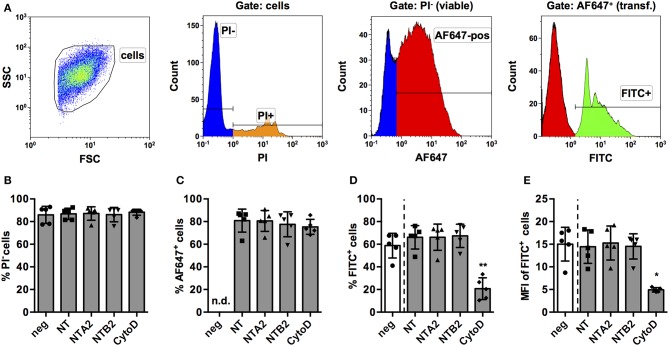
Establishment of a FACS-based assay to screen for miRNAs for effect on phagocytosis of PMA-activated THP-1 MΦ. **(A)** Activated THP-1 Mq were identified by forward scatter (FSC) and side scatter (SSC), and viable cells were selected by excluding PI^+^ cells. Viable cells were analyzed for successful transfection by positive AF647 staining of the control NT siRNA. Within the PI^−^ AF647^+^ population, phagocytic cells were identified using the FITC signal of fluorescent latex beads. Viability of cells (% of PI^−^ cells; **B**), transfection efficiency **(C)**, percentage of phagocytic cells **(D)**, and phagocytic activity (MFI of FITC^+^ cells; **E**) were measured in cells transfected with the NT siRNA alone or in combination with two non-targeting miRNAs (NTA2 and NTB2) or with cytochalasin D treatment (CytoD). In **(B–E)**, individual values of *n* = 5 independent experiments are indicated by symbols and the mean ± SD are indicated by a bar and whiskers. Statistical analysis was performed using repeated measures, one-way ANOVA. Dunnett's post analysis was used to calculate *p*-values adjusted for multiple comparisons with NT-transfected cells as control condition (*p < 0.05; **p < 0.01). In **(B)**, negative control cells (neg) were set as control condition to check if any of the treatments had an effect on viability.

Based on these results, we considered PMA-stimulated THP-1 MΦ a feasible model to screen for miRNAs that have an impact on the phagocytic activity of human macrophages. In order to further validate this model, a number of control experiments were conducted. First, we assessed if the transfection protocol had an impact on viability or phagocytosis. To this end, we transfected THP-1 MΦ with two non-targeting (NT) miRNAs from other species (NTA2 and NTB2). In order to control for transfection efficiency, a NT small interfering RNA (siRNA) labeled with AlexaFluor 647 (AF647) was co-transfected in equal concentrations with the miRNA. Additionally, cytochalasin D, a known inhibitor of actin polymerization and hence phagocytosis, was included as a functional control. The gating strategy for the screen (Figure 2A) was designed to quantify phagocytosis of opsonized latex beads as percentage and MFI of the PI^−^ (viable) AF647^+^ (transfected) FITC^+^ (containing beads) population. Generally, both viability (Figure 2B) and transfection efficiency (Figure 2C) of cells was around 80% and neither the NT siRNA, the control miRNAs, nor cytochalasin D treatment significantly reduced these parameters compared to untreated controls. However, treatment with cytochalasin D significantly reduced the percentage (Figure 2D) and MFI (Figure 2E) of the FITC^+^ population.

As a further control, scanning electron microscopy and transmission electron microscopy (SEM and TEM, respectively) were performed. In line with the inhibitory effect on actin polymerization, cytochalasin D treatment resulted in morphological changes with reduced membrane ruffling (Supplementary Figure S1A). TEM images provided clear evidence for the presence of intracellular beads in untreated cells whereas beads appeared to be stalled on the surface of cytochalasin D-treated cells (Supplementary Figure S1B). Counting of intra- and extra-cellular beads in panorama images obtained by TEM supported the notion that cytochalasin D treatment reduced the number of intracellular beads (Supplementary Figures S2, S3). Only 18.3% of the untreated MΦ contained no bead at all and most cells contained three or more beads. By contrast, the vast majority (77.4%) of cytochalasin D-treated cells contained no beads and only a small fraction of cells contained 1 or 2 beads. Collectively, TEM and SEM images indicate that the effects observed in the FACS analysis are based on the level of intracellular beads and not just due to reduced binding of beads to the cell surface. This suggests that the analysis of FITC^+^ cells in the screening protocol indeed provides data on the number and activity of phagocytic cells via the fluorescence of intracellular beads.

### High-Content Screening of a Human miRNA Library

**Figure 3:**
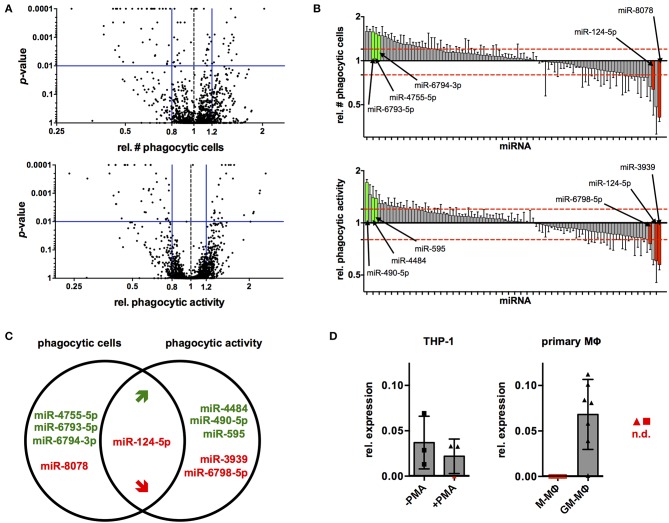
High-content screening identifies several miRNAs that alter phagocytic behavior of PMA-activated THP-1 MΦ. **(A)** Volcano plots of *p*-value over relative number (rel. #) of phagocytic cells (left panel) or relative phagocytic activity (right panel) for cells transfected with all miRNAs tested in the primary screen. Blue bars represent cutoff values for change of relative number or phagocytic activity (≤0.8 or ≥1.2) and significance (*p* ≤ 0.01). **(B)** Bar charts of rel. # of phagocytic cells (upper panel) or relative phagocytic activity (lower panel) of cells transfected with the 95 miRNAs tested in the secondary screen. Values for each miRNA are the mean ± standard deviation (SD) of triplicate measurements indicated by a bar and whiskers and were ranked according to the extent of the effect (rel. fold-change). Bars of miRNAs that met the selection criteria in both screens are labeled in green (increase) or red (decrease), and the names of the corresponding miRNAs have been connected to the bars with an arrow. For visualization of the data across all plates of the primary or secondary screen, values were normalized to the mean of the on-plate NT controls. Statistical analysis was performed on raw data of the triplicate measurements on each plate using one-way ANOVA. Dunnett's post analysis was used to calculate *p*-values adjusted for multiple comparisons with the NT set as control condition. **(C)** Venn diagram of the 10 miRNAs that robustly and reproducibly altered the number of phagocytic cells and/or phagocytic activity in the primary and secondary screen. MiRNAs that enhance phagocytosis are labeled in green, and those that reduce phagocytosis are labeled in red. **(D)** Levels of miR-124-5p transcripts in THP-1 Mo (–PMA) and MΦ (+PMA) or *ex vivo* differentiated M- and GM-Mq as assessed by qRT-PCR (*n* = 3 for THP-1 cells and *n* = 7 donors for primary cells). Symbols for samples with miR-124-5p levels below the detection limit (n.d.) are marked with red symbols.

Next, the established protocol for transfection of PMA-activated THP-1 MΦ was used to screen a library of all 2,566 currently known, human miRNAs for their effects on phagocytosis of serum-opsonized, fluorescent latex beads (Supplementary Data Sheet 1). Cut-off criteria for potential hits were changes in the proportion of phagocytic cells (% of FITC^+^ cells) and/or their phagocytic activity (MFI of FITC^+^ cells) by at least 20% with a probability of error of 1%, i.e., a relative number of phagocytic cells or MFI of ≤ 0.8 or ≥1.2 at *p* ≤ 0.01 compared to the NT controls. Using these criteria, 95 miRNAs were identified that reduced or enhanced either the percentage of FITC^+^ cells (56 miRNAs) and/or the phagocytic activity (MFI) of the FITC^+^ population (68 miRNA) or both parameters (29 miRNAs) in a primary screen (Figure 3A and Supplementary Data Sheet 1). These 95 miRNAs were tested in a secondary screen with the same stringent cut-off criteria (Supplementary Data Sheet 1 and Figure 3B). A total of 10 miRNAs met these criteria in both screens (Figure 3C). The relative number of phagocytic cells was increased by miR-4755-5p, miR-6793-5p, and miR-6794-3p and reduced by miR-8078. On the other hand, relative phagocytic activity was increased by miR-4484, miR-490-5p, and miR-595, and reduced by miR-3939 and miR-6798-5p. MiR-124-5p was the miRNA that significantly reduced both parameters.

In a next step, the endogenous levels of miR-124-5p were investigated in different macrophages using quantitative RT-PCR. In THP-1 Mo, miR-124-5p could be detected at low levels. Upon PMA activation, expression of miR-124-5p was further reduced and even dropped below the detection limit of the assay for one out of three samples (Figure 3D). Primary monocytes of healthy individuals differentiated *ex vivo* in the presence of different growth factors such as granulocyte-macrophage colony-stimulating factor (GM-CSF) or macrophage colony-stimulating factor (M-CSF) are a better model for human MΦ. *Ex vivo* differentiation of primary human Mo with GM- or M-CSF generates distinct macrophage populations that display either low (GM-MΦ) or high (M-MΦ) phagocytosis of different bacteria, viruses, and opsonized latex beads (27–30). We thus assayed miR-124-5p levels in GM-MΦ and M-MΦ (Figure 3D). In M-MΦ, miR-124-5p was below the limit of detection in all samples. By contrast, GM-MΦ of all donors analyzed contained clearly detectable levels of miR-124-5p.

### Validation of the Effects of miR-124-5p on Phagocytosis

**Figure 4:**
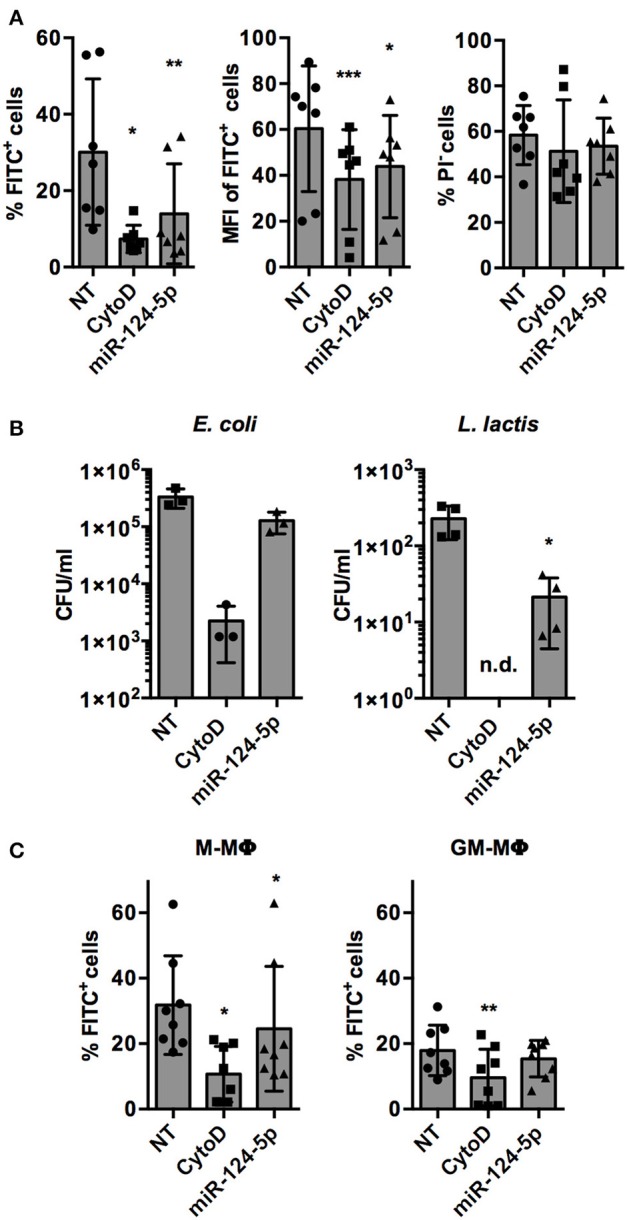
Transfection with miR-124-5p reduces phagocytosis of opsonized latex beads and live bacteria in PMA-activated THP-1 MΦ and *ex vivo* differentiated, primary M- and GM- MΦ. **(A)** Phagocytosis of FITC-labeled, opsonized latex beads by PMA-activated THP-1 MΦ treated with an NT siRNA (NT), cytochalasin D (CytoD), or miR-124-5p as percentage of phagocytic cells (% FITC^+^ cells, left), phagocytic activity (MFI of FITC^+^ cells, middle), and viability (% PI^−^ cells) across *n* = 6 independent experiments. **(B)** Phagocytosis of live *E. coli* (left panel) or *L. lactis* (right panel) in the same experimental setup assessed as intracellular colony-forming units (CFU/ml) by gentamicin protection assay (*n* = 3–4 independent experiments). **(C)** Phagocytosis of FITC-labeled, opsonized latex beads by *ex vivo* differentiated M-MΦ (left panel) and GM-MΦ (right panel) with the indicated treatments as percentage of phagocytic cells (% FITC^+^ cells; *n* = 8 donors). In all panels, values from each experiment are indicated by symbols and the mean ± SD are indicated by a bar and whiskers (n.d.: not detected). Statistical analysis was performed using repeated measures, one-way ANOVA with Geisser–Greenhouse correction. Dunnett's post analysis was used to calculate *p*-values adjusted for multiple comparisons with the NT set as control condition (*p < 0.05; **p < 0.01; ***p < 0.001).

The only miRNA that reproducibly and significantly reduced both parameters was miR-124-5p. Thus, the observed effects of miR-124-5p were validated and analyzed in more detail in further experiments. This confirmed the results of the screen. Upon transfection with miR-124-5p, the percentage of FITC^+^ cells was reduced significantly compared to NT-transfected cells (13.9 ± 13.1% vs. 30.1 ± 19.2%; *p* = 0.002; Figure 4A). Likewise, the number of phagocytic cells was also reduced significantly by cytochalasin D treatment (7.4 ± 3.6%; *p* = 0.020). Similarly, MFI was reduced significantly by both cytochalasin D (38.2 ± 21.7; *p* < 0.001) and miR-124-5p transfection (43.9 ± 22.3; *p* = 0.015) compared to NT-transfected cells (60.4 ± 27.4). The percentage of PI^−^ cells was not affected by treatment with miR-124-5p or cytochalasin D (Figure 4A). However, a slight, yet significant increase in PI signal intensity was observed in PI^−^ cells treated with miR-124-5p (MFI 3.0 ± 1.7) compared to the NT-treated controls (MFI 1.6 ± 0.8; *p* = 0.013), suggesting that miR-124-5p might also have a slight pro-apoptotic effect.

To further validate the data of the screens, additional experiments were performed for two other miRNAs identified in the screens: miR-8078 and miR-6793-5p. Both miRNAs also had robust and reproducible effects (Supplementary Figure S4). While miR-8078 significantly reduced the percentage of FITC^+^ cells and wells as their MFI, miR-6793-5p had the opposite effect and significantly increased both parameters compared to untreated controls, confirming the results of the screens.

Phagocytosis of opsonized latex beads is mediated by complement and F_c_γ receptors (1). In order to test if miR-124-5p has similar effects on phagocytic pathways mediated by other receptors, phagocytosis of (non-opsonized) live bacteria was assessed (Figure 4B). Gentamycin protection assays demonstrated that the phagocytosis of Gram-negative (*Escherichia coli*; 1.3 ± 0.5 × 10^5^ vs. 3.3 ± 1.2 × 10^5^ colony-forming units (CFU)/well) and Gram-positive (*Lactococcus lactis*, 2.1 ± 1.7 × 10^1^ vs. 2.3 ± 1.1 × 10^2^ CFU/well) bacteria was reduced in cells treated with miR-124-5p compared to the NT control cells. This effect was statistically significant for *L. lactis* (*p* = 0.033). As expected, treatment with cytochalasin D significantly reduced intracellular bacteria for both *E. coli* and *L. lactis*.

Additionally, we performed phagocytosis assays in M- and GM-MΦ transfected with miR-124-5p (Figure 4C). As observed previously (29), M-MΦ displayed higher phagocytosis of opsonized latex beads than GM-MΦ. Moreover, in M-MΦ, i.e., the cell type that did not express miR-124-5p endogenously, miR-124-5p transfection significantly reduced the percentage of FITC^+^ cells compared to the NT controls (24.6 ± 19.1% vs. 31.8 ± 15.1%; *p* = 0.043). In both populations, phagocytosis was significantly reduced upon cytochalasin D treatment. Thus, the expression pattern and results from experiments in transfected primary MΦ are in line with a suppressive effect of miR-124-5p on phagocytosis.

### Identification of Potential Target Genes of miR-124-5p

*In silico* analyses using the miRWalk2.0 suggested a total of 57,204 sites in 17,236 genes as potential targets of miR-124-5p (Supplementary Data Sheet 2). Pathway analysis revealed that the top three KEGG pathways in which potential target genes were significantly enriched were “pathways in cancer,” “Wnt signaling pathway,” and “regulation of actin cytoskeleton” (Supplementary Data Sheet 2).

**Figure 5:**
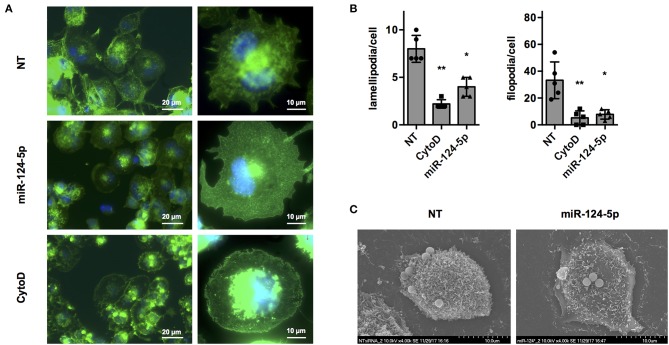
Transfection with miR-124-5p alters cell morphology of PMA-activated THP-1 MΦ. **(A)** Fluorescence microscopy of PMA-activated THP-1 MΦ following the indicated treatments. Actin cytoskeleton was stained with Alexa Fluor 488-phalloidin and nuclei with Hoechst 33342. Images were acquired with a Zeiss AxioObserver Z1 using a 40× (left panels; scale bars: 20 μm) or a 100× objective (right panels; scale bars: 10 μm) and overlays of the fluorescence channels for phalloidin (green) and Hoechst (blue) are shown. **(B)** Quantitative analysis of lamellipodia and filopodia formed by PMA-activated THP-1 MΦ following the indicated treatments. Image analysis was performed as described in Supplementary Figure S5. A total of 47–57 cells (depending on the condition) of *n* = 5 independent experiments were analyzed. Mean values for each experiment are indicated by symbols and the mean ± SD across experiments are indicated by a bar and whiskers. Statistical analysis was performed using repeated measures, one-way ANOVA with Geisser–Greenhouse correction. Dunnett's post analysis was used to calculate *p*-values adjusted for multiple comparisons with the NT set as control condition (*p < 0.05; **p < 0.01). **(C)** SEM of PMA-activated THP-1 MΦ following the indicated treatments. Images were acquired with a Hitachi S-5200 scanning electron microscope at 4,000 × magnification. Representative cells of *n* = 3 independent experiments are shown.

Regulation of the actin cytoskeleton is crucial for most if not all cellular functions of macrophages including formation of pseudo- and filopodia, uptake of bacteria, apoptotic cells and other particles, intracellular trafficking of vacuoles, and migration (31). In order to get a first indication if miR-124-5p affects organization of the actin cytoskeleton, fluorescence, and scanning electron microscopy (SEM) were performed (Figure 5). Fluorescence microscopy of cells stained with AlexaFluor 488-labeled phalloidin (Figure 5A) indicated that transfection with miR-124-5p resulted in a phenotype similar to that observed with cytochalasin D treatment with more rounded cells, less structured actin filaments, and markedly reduced formation of cellular protrusions. Quantitative image analysis revealed a significantly reduced number of lamellipodia and filopodia in cells treated with miR-124-5p compared to NT-treated controls (Figure 5B and Supplementary Figure S5). These observations were supported by SEM (Figure 5C). Here, cells treated with miR-124-5p appeared to have a by far smoother surface compared to NT-treated cells. Thus, both fluorescence microscopy and SEM indicate that miR-124-5p affects organization of the actin cytoskeleton.

**Figure 6:**
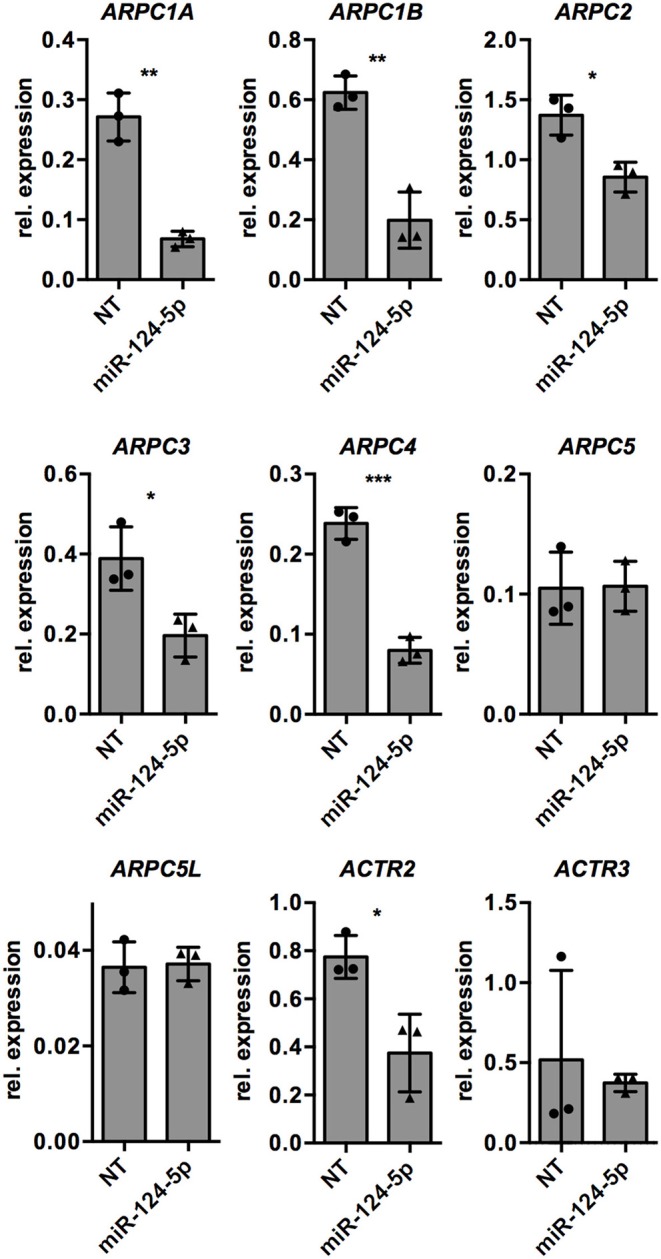
Transfection with miR-124-5p alters expression of potential target genes in PMA-activated THP-1 MΦ. Expression levels of genes encoding the subunits of the ARP2/3 complex in PMA-activated THP-1 MΦ transfected with either the NT siRNA or miR-124-5p (*n* = 3 independent experiments). Values from each experiment are indicated by symbols and the mean ± SD are indicated by a bar and whiskers. Statistical analysis was performed using Student's *t*-test (*p < 0.05; **p < 0.01; ***p < 0.001).

Since formation and dynamics of the actin cytoskeleton are directly regulated by the ARP2/3 complex, we manually searched the list of potential target genes of miR-124-5p for the genes encoding the subunits of the ARP2/3 complex. Indeed, the genes for all subunits of the complex are predicted targets of miR-124-5p. We thus performed transcriptional analyses by qRT-PCR on these genes (Figure 6). This revealed that transcript levels of *ARPC1A, ARPC1B, ARPC2, ARPC3, ARPC4*, and *ACTR2* were significantly reduced in cells treated with miR-124-5p compared to NT controls, suggesting that these genes might be targets of miR-124-5p.

**Figure 7:**
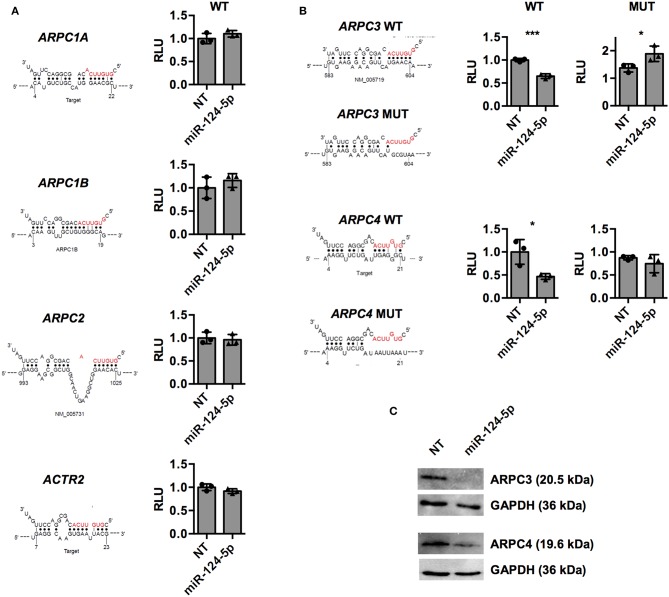
Dual luciferase assays indicate that miR-124-5p directly binds to predicted target sequences in *ARPC3* and *ARPC4* and reduces corresponding protein levels in PMA-activated THP-1 MΦ. **(A,B)** Relative *Renilla* luciferase activity in HEK293 cells co-transfected with miR-124-5p or NT siRNA and dual luciferase reporter plasmids with predicted wildtype (WT) binding sequences in the *ARPC1A, ARPC1B, ARPC2, ARPC3, ARPC4*, and *ACTR2* mRNAs. **(B)** For binding sites of genes *ARPC3* and *ARPC4* yielding reduced relative luminescence units (RLUs) indicating direct interaction with miR-124-5p, additional dual luciferase reporter plasmids with mutated (MUT) binding sequences were tested. Values are RLUs of the firefly luciferase normalized for transfection efficiency using the constitutive *Renilla* luciferase activity. Statistical analysis was performed using Student's *t*-test (*p < 0.05;***p < 0.001). **(C)** Western blot for ARPC3 or ARPC4 protein levels. One representative blot of *n* = 3 independent experiments is shown.

To further investigate direct regulation of the components of the ARP2/3 complex, predicted binding sites of miR-124-5p in mRNAs of *ARPC1A, ARPC1B, ARPC2, ARPC3, ARPC4*, and *ACTR2* were retrieved from the respective databases of miRWalk 2.0 (32) (Supplementary Table S1 and Figure 7). These binding sequences were cloned into the 3′UTR of the *luc* gene on a dual luciferase report plasmid and the resulting constructs were transfected into HEK-293T cells in combination with either miR-124-5p or the NT siRNA. For the predicted binding sequences of *ARPC1A, ARPC1B, ARPC2*, and *ACTR2*, no difference in the firefly luciferase activity was observed between cells transfected with miR-124-5p or NT siRNA, suggesting that these sequences are not targeted by miR-124-5p (Figure 7A). By contrast, reduced firefly luciferase activity was observed in cells co-transfected with the constructs containing the predicted binding sites of *ARPC3* and *ARPC4* and miR-124-5p and compared to NT controls (Figure 7B). This suggests that miR-124-5p may directly regulate expression of these genes via the cloned binding sites. Further dual luciferase reporter assays with mutated binding sequences confirmed the specificity of the interaction of miR-124-5p with this sequence (Figure 7B). Additionally, Western blot analysis revealed that ARPC3 and ARPC4 protein levels were reduced in PMA-activated THP-1 cells transfected with miR-124-5p compared to cells transfected with the NT siRNA (Figure 7C).

## Discussion

Over the last years, the crucial role of miRNAs in the regulation of immune responses and macrophage function and polarization has been increasingly appreciated (33–36). However, most studies have analyzed transcriptional levels of miRNAs in Mo and different subpopulations of *ex vivo* generated primary MΦ to define their role during differentiation (11–13, 15, 37). Regarding the role of macrophages in clearance of infections, it has been shown that toll-like receptor (TLR) signaling triggers differential expression of various miRNAs and different miRNAs target TLR gene expression (35). However, there are only a few reports on individual miRNAs and their impact on phagocytosis. For example, miR-21 expression is enhanced in murine bone marrow-derived MΦ (BMDM) following infection with *Listeria monocytogenes* and BMDM of miR-21^−/−^ mice show enhanced uptake of *L. monocytogenes*, FITC-labeled *E. coli* bioparticles, and FITC-dextran (38). Similarly, miR-142-3p expression is transiently upregulated during *Mycobacterium smegmatis* infection of murine J774 (10). Transfection with an miR-142-3p mimic reduced phagocytic uptake of *M. smegmatis* and *M. tuberculosis* whereas an miR-142-3p inhibitor had the opposite effect. Likewise, overexpression of miR-615-5p increased phagocytic uptake of FITC-*E. coli* by murine splenic MΦ (39). Conversely, bacterial infection and LPS stimulation enhance expression of miR-15a and miR-16 in BMDM but myeloid-specific deletion of the locus encoding miR-15a/16 resulted in increased phagocytosis of FITC-labeled *E. coli* due to increased expression of the LPS receptor TLR-4 (40). Naqvi et al. followed up on miR-24, miR-30b, and miR-142-3p, which were identified by transcriptional profiling as differentially expressed in Mo, M-MΦ, and GM-MΦ (11). Transfection of Mo, M-MΦ, and GM-MΦ with these miRNAs led to reduced phagocytosis of rhodamine-labeled, Gram-positive and Gram-negative bioparticles as well as intact *E. coli* bacteria and IgG-opsonized latex beads (41, 42). Nevertheless, a systematic, functional identification of miRNAs that modulate phagocytosis has, to the best of our knowledge, not been performed.

To this end, we have established a system to screen the entire human miRNome for miRNAs modulating phagocytosis using PMA-activated THP-1 MΦ. Using this system, we identified a total of 10 miRNAs that consistently altered the phagocytic behavior. For all three miRNAs tested in further experiments (miR-124-5p, miR-8087, and miR-6793-5p), the results of the screen could be confirmed (Figure 4 and Supplementary Figure S4), demonstrating that the screening system and hit identification established provide robust results. Surprisingly, none of these miRNAs has been known or analyzed for a role in phagocytosis. Most of the identified miRNAs have been linked with cancer. MiR-4755-5p and miR-8078 were suggested as oncomirs (43, 44), and miR-6794-5p, and miR4484 were shown to act as tumor suppressors (45–47). Interestingly, miR-490-5p and miR-595 were reported to promote or suppress tumor growth depending on the type of cancer (48, 49). MiRNAs miR-6793-5p and miR-3939 were shown to be increased in diabetic patients (50, 51). MiR-6798-5p has not been linked with any phenotype or role so far.

The fact that none of the identified miRNAs were linked to phagocytosis in previous studies may be due to several reasons. On the one hand, this might be related to the cellular model systems. The effects of miRNAs on their target mRNAs strongly depend on the cell type analyzed (52). Thus, it is not surprising that a functional screen of the effects of miRNAs on phagocytosis in PMA-activated THP-1 cells yields different miRNAs compared to transcriptional profiling studies in human monocyte-derived MΦ or murine BMDM populations differentiated *ex vivo* (11, 12, 15, 37). On the other hand, effects of miRNAs are dose-dependent (53, 54). While in most of the studies cited above, miRNA mimics were used at concentrations of 50–100 nM to produce an effect (10, 41, 42), we have used a relatively low miRNA concentration (15 nM) in our screen to avoid off-target effects or saturation of the RNAi machinery. Finally, the optimal time point to detect an effect of a miRNA may vary depending on, e.g., miRNA degradation and turnover transcriptional levels and regulation of the target gene or stability of the respective proteins (55–58). Thus, it is possible that miRNAs previously shown to modulate phagocytosis in other model will produce an effect in PMA-activated THP-1 MΦ, when assayed at other time points or concentrations. For the same reasons, it is quite likely that other, additional miRNAs may be identified if the screening experiments would have been performed at different miRNA concentrations or at time points post-transfection. It is possible that in such experiments, some of those miRNA would emerge as good candidates that have already shown effects in the presented experiments, but effects were not robust enough to meet cut-off criteria in both screens.

Among the miRNAs that emerged from our screening strategy, miR-124-5p was the only miRNA that consistently and significantly reduced both the number of phagocytic cells as well as the phagocytic activity of PMA-activated THP-1 MΦ. Previously, miR-124-5p was shown to be highly expressed in cells of the nervous system of humans and mice (59). Also, low or absent expression of miR-124-5p is associated with glioblastoma and colorectal cancer (60, 61). To the best of our knowledge, our results represent the first report on a role for miR-124-5p in regulation of phagocytosis of human MΦ. The results of the screen were corroborated and extended in further experiments showing that transfection of primary MΦ with miR-124-5p also reduced phagocytosis of opsonized latex beads. This effect was more pronounced in M-MΦ, which do not express detectable levels of miR-124-5p, compared to GM-MΦ that express miR-124-5p. Thus, expression of miR-124-5p may have a suppressive effect on phagocytosis in macrophages, although it may not be the only factor responsible for the different phagocytic behavior of M- and GM-MΦ. Formal demonstration of the suppressive effect of miR-124-5p on phagocytosis and a role of this mechanism *in vivo* will certainly require further experiments. As a working hypothesis for these experiments, we would propose a model in which miR-124-5p expression is low in M-MΦ, which is the prevailing phenotype in tissue homeostasis. In this model, activation by inflammatory stimuli and differentiation to inflammatory GM-MΦ induces miR124-5p expression, thereby reducing phagocytic activity in favor of an enhanced capacity to kill phagocytosed pathogens.

The effect of miR-124-5p was observed with opsonized latex beads, which are recognized by opsonic receptors, and non-opsonized, intact cells of the Gram-positive bacterium *L. lactis*. Also, a slight reduction in phagocytosis of non-opsonized *E. coli* cells was observed, although this effect was not statistically significant. Bacterial cells as well as lipopolysaccharide, a component of the outer membrane of Gram-negatives, trigger various signaling cascades in MΦ (1) and were shown to alter expression of different miRNAs (10, 40, 62). Thus, it is possible that the signaling cascades and events triggered by *E. coli* interfere with or override the effects of miR-124-5p while those triggered by *L. lactis* do not or do so only to a minor extent.

Nevertheless, non-opsonized bacteria are internalized via non-opsonic receptors and opsonized latex beads by opsonic receptors (1). As a reduction on phagocytosis by miR-124-5p was observed in both cases, we reasoned that miR-124-5p may act independent of the receptors on a more central event. Microscopic analyses indicated that transfection with miR-124-5p leads to changes in the actin cytoskeleton. Using *in silico* analyses, genes for different subunits of the ARP2/3 complex were identified as potential targets of miR-124-5p and reduced expression of six of these genes was observed following transfection with miR-124-5p. The ARP2/3 complex is a crucial regulator of the dynamics of the actin cytoskeleton and consists of seven subunits (63). ARPC1–ARPC5 are structural proteins that interact with existing actin mother filaments, while ARP2 and ARP3 (encoded by *ACTR2* and *ACTR3*, respectively) are required for polymerization/growth of branching daughter filaments (7). Together with different context-related nucleation promoting factors, ARP2/3-mediated actin polymerization drives formation of membrane ruffles, lamellipodia, filopodia, and phagocytic structures (31).

Potential miR-124-5p binding sites were predicted in all target mRNAs by different databases of MirWalk2.0 (Supplementary Table S1). The predicted sites for *ARPC3* and *ARPC4* were confirmed experimentally by luciferase assays indicating that these genes may be direct targets of miR-124-5p. Additionally, reduced levels of the corresponding proteins were observed after transfection with miR-124-5p, providing a mechanistic explanation for the observed changes in actin cytoskeleton, phagocytic activity, and its receptor independent effects on phagocytosis. The algorithms used by databases to predict miRNA binding motifs differ. Thus, it cannot be excluded that the other genes that show reduced expression upon transfection are also direct targets of miR-124-5p via binding sites that may be predicted using other bioinformatic tools such as, e.g., the STarMir online tool (64). Alternatively, these genes could also be subject to an indirect regulation by, e.g., transcription factors targeted by miR-124-5p.

Previous studies have also shown effects of miRNAs on regulators of actin cytoskeleton dynamics. For instance, miR-143 and miR-145 control cytoskeletal dynamics in smooth muscle cells by targeting myocardin-related transcription factors that regulate remodeling of the cytoskeleton (65, 66). Expression levels of miR-205 have an impact on the migratory capacity of keratinocytes by regulating the lipid phosphatase SHIP2 (67), a known regulator of actin organization (68, 69). MiR-200 directly targets the 3′UTR of WAVE3, which is an important actin nucleation promoting factor affecting actin remodeling and motility of cancer cells, and high miR-200 levels are correlated with an invasive phenotype of cancer cell lines (70–72). With respect to phagocytosis, miR-142-3p has been shown to target the actin nucleation promoting factor N-WASP that activates the ARP2/3 complex, thereby enhancing phagocytic uptake of mycobacteria (10). Here, we demonstrate that miR-124-5p modulates phagocytosis of MΦ by directly regulating expression of different subunits of the ARP2/3 complex representing the most central point of regulation of the actin cytoskeleton by a miRNA described so far.

## Materials and Methods

### Cultivation of Eukaryotic Cells

THP-1 cells (ATCC® TIB-202) were routinely cultivated in RPMI 1640 (Sigma) complemented with 10% (v/v) fetal calf serum (FCS; Gibco), 1% (v/v) non-essential amino acids, 1% (v/v) L-glutamine, and 1% penicillin–streptomycin (stock solution: 10,000 units/ml penicillin and 10 mg/ml streptomycin; Sigma) under standard cell culture conditions (37°C, 5% CO_2_). For experiments, THP-1 cells were seeded at a density of 5 × 10^4^ per well in 96-well tissue culture plates in 100 μl of the same medium containing 100 nM PMA (Sigma) for 72 h to induce a macrophage-like phenotype.

HEK293 cells (ATCC® CRL-1573) were routinely cultivated in high glucose DMEM (Sigma) complemented with 10% (v/v) FCS, 1% (v/v) non-essential amino acids, 1% (v/v) L-glutamine, and 1% (v/v) penicillin/streptomycin in T75 cell culture flasks under standard cell culture conditions.

Monocytes were isolated from buffy coats of anonymous healthy donors obtained from a local blood bank. All experiments were approved by the Institutional Review Board of the University of Ulm and informed written consent was obtained from all donors approving and authorizing the use of their material for research purposes. Peripheral blood mononuclear cells were isolated by density gradient centrifugation using Histopaque®-1077 (Sigma) as described previously (29) and 4–5 × 10^7^ cells were seeded into T25 cell culture flasks and incubated 1 h in RPMI 1640 containing 1% human serum (Sigma) at 37°C and 5% CO_2_. Non-adherent cells were removed by washing twice with PBS (Sigma). Adherent monocytes were allowed to differentiate in RPMI 1640 containing 10% (v/v) FCS, 1% (v/v) non-essential amino acids, 1% (v/v) L-glutamine, 1% (v/v) penicillin/streptomycin, and either 10 ng/ml GM-CSF (Peprotech) or 30 ng/ml M-CSF (R&D Systems) for 7 days.

### miRNA Library and Transfection Protocol

The human MISSION® microRNA Mimic Library (miRBase, version 21) was purchased from Sigma (Sigma). This library contains synthetic miRNA mimics of 2,752 annotated miRNAs according to miRbase v.21 and two NT control miRNAs of non-human origin (NTA2 from *Arabidopsis thaliana* and NTB2 from *Caenorhabditis elegans*). Some miRNAs in the library had more than one accession ID but identical sequences. These miRNAs were only tested once, resulting in a total of 2,566 miRNAs analyzed. For transfection, PMA-activated THP-1 MΦ, primary MΦ, or HEK cells were incubated for 72 h in 100 μl of serum-reduced medium Opti-MEM (ThermoFisher Scientific) with 0.2 μl of Lipofectamine RNAiMAX (Invitrogen) mixed with individual miRNA mimics (final concentration, 15 nM per well) or control RNAs according to the manufacturer's instructions. As a transfection control, cells were co-transfected with NT AllStars negative control siRNA labeled with AF647 (Qiagen).

### Phagocytosis of Fluorescent Latex Beads

Phagocytosis was assayed using FITC-labeled latex beads (diameter 2 μm; Sigma), which were opsonized by incubation in RPMI 1640 medium with 20% (v/v) human AB serum (Sigma) for 30 min at 37°C. After 72 h of transfection with miRNAs or controls, 5 × 10^5^ opsonized beads (i.e., 20 μl of beads in AB serum) were added to 5 × 10^4^ cells in the 96-well plates (see above), i.e., a multiplicity of infection (MOI) of 10 beads per cell, and incubated for 1 h at 37°C. Afterwards, cells were detached from plates by using Trypsin-EDTA (Sigma) for 20 min at 37°C and stained with 10 μg/ml PI (Sigma) in RPMI 1640 medium including 0.1% (v/v) anti-clumping agent (Gibco) and analyzed using flow cytometry.

### Bacterial Strains and Gentamicin Protection Assays

For gentamicin protection assays, *E. coli* D2241 (73) and *L. lactis* MG1363 (74) were used. *E. coli* was cultivated in lysogeny broth at 37°C with aeration on an orbital shaker and *L. lactis* was cultivated in M17 agar or medium supplemented with 1% (w/v) glucose at 30°C without agitation.

Phagocytosis of bacteria by MΦ was measured by gentamicin protection assay as described previously (75) with minor modifications. Briefly, bacteria of o/N cultures were washed once in PBS and adjusted to an OD_600_ of 0.01 (i.e., 8 × 10^6^ CFU/ml for *E. coli* and 6 × 10^6^ CFU/ml for *L. lactis*) in RPMI 1640 medium without antibiotics. Subsequently, THP-1 MΦ were infected with bacteria at an MOI of 20 bacteria per cell. To facilitate sedimentation and contact with macrophages, bacteria were centrifuged onto the cells at 300 × *g* for 1 min and phagocytosis was allowed during 1 h at 37°C and 5% CO_2_. Afterwards, gentamicin (final concentration, 10 μg/ml, Sigma) was added to the cells to allow elimination of non-phagocytosed extracellular bacteria. Then, cells were washed with PBS prior to 5 min incubation with 0.1% (v/v) Triton-X100® (Sigma) at 4°C in order to disrupt cells. Lysates were collected and 10-fold serial dilutions were prepared in PBS. Of each dilution, six 10 μl samples were spot-plated on the corresponding agar and incubated 24 h at the abovementioned optimal temperature. On the next day, CFU were counted and calculated per milliliter. Cytochalasin D treatment was applied to cells 30 min prior to incubation with bacteria as a functional control to inhibit phagocytosis.

### Flow Cytometry and Data Analysis

Flow cytometry for the primary and validation screen was performed on a MACSQuant® Analyzer 10 (Miltenyi Biotec, Bergisch Gladbach, Germany) equipped for automated sampling from 96-well cell culture plates and on a FACSCalibur (Becton-Dickinson) for all other experiments. FACS data from the primary and validation screen were analyzed using the MACSQuantify Software 2.0 (Miltenyi Biotec, Bergisch Gladbach, Germany), while for all other experiments, the Kaluza Analysis software (version 2.1, Beckman Coulter) was used.

Cells were identified according to size (forward scatter) and granularity (side scatter) to exclude cell debris and free latex beads from further analysis. The PI signal was recorded in the entire cell population to exclude PI^+^ necrotic cells, and PI^−^ cells were selected and further analyzed for successful transfection by positive AF647^+^ staining of the co-transfected NT control siRNA. Only PI^−^ AF647^+^ cells were analyzed for FITC fluorescence intensity of the fluorescent latex beads (Figure 2A). Final readouts for phagocytosis were (a) the proportion (percentage) of viable, phagocytic cells and (b) the MFI of this population, which is a (semi)quantitative measure for the amount of beads taken up (i.e., phagocytic activity of these cells). To allow comparisons and visualization of data across all plates of the screen in one graph, values were normalized to the mean of the on-plate NT controls, which was set to 1. The resulting values are relative numbers of phagocytic cells or relative phagocytic activity.

### Microscopic Analyses

Samples for SEM were fixated and prepared as described previously (76). Samples were then analyzed on a Hitachi S-5200 scanning electron microscope (Hitachi High-Technologies Corporation) with an acceleration voltage of the electron beam of 10.0 kV and an emission current of 10 μA.

For TEM imaging, cells were grown on sterile sapphire discs placed inside wells of a tissue culture plate and treated as described above and then processed by high-pressure freezing as described (77). TEM was conducted with a JEM-1400 electron microscope (Jeol) at an electron beam acceleration voltage of 120 kV and the pictures were taken and stored by the inherent software iTEM (Olympus Imaging Solution GmbH).

In order to analyze the actin cytoskeleton by fluorescence microscopy, cells were differentiated in Nunc™ Lab-Tek chamber slides (ThermoFisher Scientific). After differentiation and transfection, cells were washed once with PBS and then fixed in a 4% (w/v) paraformaldehyde (Sigma) in PBS solution for 15 min at 37°C prior to staining. After fixation, cells were washed three times with PBS and incubated for 25 min at room temperature in the dark in PBS containing 0.5% (w/v) saponin (Sigma), Alexa Fluor 488 (AF488) phalloidin (Invitrogen, dilution 1:500), and Hoechst 33342 (Invitrogen, dilution 1:10.000). Finally, cells were again washed with PBS and imaged using an AxioObserver Z1 microscope (Zeiss). The software ZEN 2012 (Zeiss) was used for image capture and analysis.

### *In silico* Analyses

Potential target genes regulated by miR-124-5p and the corresponding binding sequences were identified by *in silico* analyses. Target genes were predicted using the MicroRNA-gene targets module of the miRWalk2.0 online tool (32) with default settings and all 12 possible databases included. Binding sites of miR-124-5p in potential target genes were retrieved from the respective databases. Potential binding sites of miR-124-5p in genes for the ARP2/3 subunits were modeled using the STarMir online tool (64) on the S-fold web server (http://sfold.wadsworth.org).

### RNA Isolation and qRT-PCR

Total RNA was isolated and purified from at least 1 × 10^6^ cells using the RNeasy Mini Kit (Qiagen) following the manufacturer's instructions. DNaseI treatment (ThermoFisher Scientific) was performed to remove residual genomic DNA, and cDNA was synthesized using the ImProm-II Reverse Transcription System Kit (Promega). Expression levels of potential miR-124-5p target mRNAs were quantified by qRT-PCR using the MESA BLUE qPCR MasterMix for SYBR® Assay (Eurogentec) and target-specific primer pairs (Supplementary Table S2). All qRT-PCR primers were selected from PrimerBank (78).

MiRNAs were isolated and purified from at least 1 × 10^6^ cells using the miRNeasy Mini Kit (Qiagen) following the manufacturer's instructions. Synthesis of cDNA from miRNAs and quantification of miRNA transcript levels were performed using miRCURY LNA miRNA PCR Starter Kit (Qiagen) according to the manufacturer's instructions. The kit included specific and validated primers for miR-124-5p and miR-93-3p, which was used as control miRNA for normalization.

PCR cycling was performed on a CFX96 Touch™ real-time PCR detection system (Bio-Rad) and data was analyzed using the CFX Manager® software (Version 3.1; Bio-Rad). Relative gene expression was determined by normalization to *GAPDH* expression levels and the 2^(−Δ*CT*)^ method.

### Dual Luciferase Reporter Assays

Interaction of miR-124-5p with predicted binding sites in target mRNAs was probed using the pmirGLO® Dual Luciferase miRNA target expression vector (Promega). Potential binding sites were cloned into the 3′UTR of the firefly luciferase reporter gene encoded on pmirGLO® by Gibson Assembly (79). For binding sites that yielded positive results indicating direct interaction with miR-124-5p (i.e., a reduction in luciferase activity), mutated binding sites were created by replacing complementary nucleotides of the seed sequence of miR-124-5p with nucleotides identical to the suspected target sequence. For cloning, the pmirGLO® vector was linearized by digestion with KpnI/BglII. For each binding site, two PCR fragments were generated by target-specific primer pairs (Supplementary Table S3). The PCR products contained the potential binding sequences and either an upstream or a downstream flanking sequence homologous to the pmirGLO® vector backbone. Subsequently, the two DNA fragments were incorporated into pmirGLO® in an isothermal reaction using the Gibson Assembly Master Mix (NEB) and transformed into *E. coli* DH10B according to a standard protocol.

For dual luciferase assays, 100 ng of a dual luciferase vector harboring one of the predicted binding sites of miR-124-5p and 20 nM of miR-124-5p or NT siRNA were co-transfected into HEK293 cells using Lipofectamine® 3000 (ThermoFisher Scientific) for the plasmid and Lipofectamine RNAiMax (ThermoFisher Scientific) for mi/siRNAs, respectively. After 72 h, luciferase activity was quantified using the Dual-Glo® Luciferase Assay System (Promega) in a microplate reader (Tecan). The *Renilla* luciferase reporter also encoded on the pmirGLO® vector served as a control to ensure equal transfection efficiencies. To obtain relative firefly luciferase activity, the ratio between the firefly luminescence and the *Renilla* luminescence was calculated per well and each sample was then normalized to the mean of cells transfected with the NT control RNA.

### SDS-PAGE and Western Blot

Cell lysates from at least 1 × 10^6^ cells were prepared for protein analysis. For extraction of cytoskeletal proteins, cells were detached from culture plates by incubation with Trypsin–EDTA (Sigma) for 20 min at 37°C and then pelleted by centrifugation for 4 min at 350 × *g*. Supernatant was removed and cells were incubated in Tris/Triton buffer containing 10 mM Tris (pH 7.4), 100 nM NaCl, 1 mM EDTA, 1 mM EGTA, 1% (v/v) Triton X-10, 10% (v/v) glycerol, 0.1% (v/v) SDS, and 0.5% (v/v) deoxycholate for 20 min. Samples were centrifuged for 30 min at 10,000 × *g* to precipitate cell debris and the protein concentration of the supernatants was determined by Pierce® BCA Protein Assay Kit (ThermoFisher Scientific). For SDS-PAGE, 50 μg of total protein was mixed with Laemmli buffer, incubated at 95°C for 10 min, and run on 12% polyacrylamide gels. Following electrophoresis, proteins were electroblotted on a nitrocellulose membrane (Macherey-Nagel) and membranes were blocked in 5% (w/v) BSA solution in TBS-T buffer for 1 h. Hybridization with mouse anti-ARPC3 (BD Transduction Laboratories™), rabbit anti-ARPC4 (Sigma-Aldrich), or mouse anti-GAPDH (Epitope Biotech; both antibodies diluted 1:500) in 5% (w/v) BSA in TBS-T was performed overnight at 4°C. Membranes were washed three times before incubation with goat anti-mouse or anti-rabbit HRP-conjugated antibody (ThermoFisher Scientific) diluted 1:5000 in 5% (w/v) BSA in TBS-T for 1 h at room temperature. After three further washings in TBS-T (10 min each), membranes were overlaid with Pierce® ECL Western Blotting Substrate (ThermoFisher Scientific) and chemiluminescence signals were detected using an iBright FL1000 visualization system (Invitrogen). Protein expression was semi-quantitatively determined using the ImageJ software tool (80).

### Statistical Analysis

Statistical analysis was performed using GraphPad Prism (version 7; GraphPad Software Inc.). Data of experimental setups with more than two comparison groups were analyzed with one-way analysis of variance (ANOVA) and Dunnett's *post-hoc* tests for multiple comparisons. The same analysis was used to identify miRNAs with significant effects on the proportion of phagocytic cells and the phagocytic activity compared to NT controls on individual plates of the screen using the raw data of the FACS analysis. For inter-plate data comparison, each miRNA value was normalized to the mean of the corresponding on-plate NT siRNA control. Where appropriate and indicated, Geisser–Greenhouse correction for repeated measures was performed. Data of experimental setups with two experimental groups were analyzed using two-tailed Student's *t*-test. Differences between experimental groups with a confidence level of 95% (*p* < 0.05) were considered statistically significant.

## Data Availability Statement

The datasets generated for this study are available on request to the corresponding author.

## Ethics Statement

The studies involving human participants were reviewed and approved by Institutional Review Board of the University of Ulm. The patients/participants provided their written informed consent to participate in this study.

## Author Contributions

CR, KO, and JT conceived the study. EH, TB, NW, SF, RR, AR, and MK carried out the experiments. EH, PC, RH, PW, and CR analyzed the data. EH, PC, and CR drafted the manuscript. All authors contributed to preparing the final version of the manuscript and read and approved the final manuscript.

### Conflict of Interest

The authors declare that the research was conducted in the absence of any commercial or financial relationships that could be construed as a potential conflict of interest.
