# Supplementary material for: mir-124-5p Regulates Phagocytosis of Human Macrophages by Targeting the Actin Cytoskeleton via the ARP2/3 Complex
Source: Front Immunol. 2019 Oct 4;10:2210. doi: 10.3389/fimmu.2019.02210 (PMC6787173; doi:10.3389/fimmu.2019.02210)
Supplement: Supplementary file 1 [file Presentation_1.pdf]

Supplementary Figures and Tables

miR-124-5p regulates phagocytosis of human macrophages by targeting the actin cytoskeleton via the ARP2/3 complex

Estefania Herdoiza Padilla[1], Peter Crauwels[1], Tim Bergner[2], Nicole Wiederspohn[1], Sabrina Förstner[1], Rebecca Rinas[1], Anna Ruf[1], Michael Kleemann[3], Rene Handrick[3], Jan Tuckermann[4], Kerstin Otte[3], Paul Walther[2], and Christian U. Riedel[1,*]

[1] Institute of Microbiology and Biotechnology, University of Ulm, Germany

[2] Central Facility for Electron Microscopy Facility, Ulm University, Germany

[3] Institute of Applied Biotechnology, University of Applied Sciences Biberach, Germany

[4] Institute of Comparative Molecular Endocrinology, Ulm University, Ulm, Germany

* Corresponding author. E-mail: christian.riedel@uni-ulm.de; Phone: +49 (0)731 502 24853.

ORCID: 0000-0001-7134-7085

Supplementary Figures

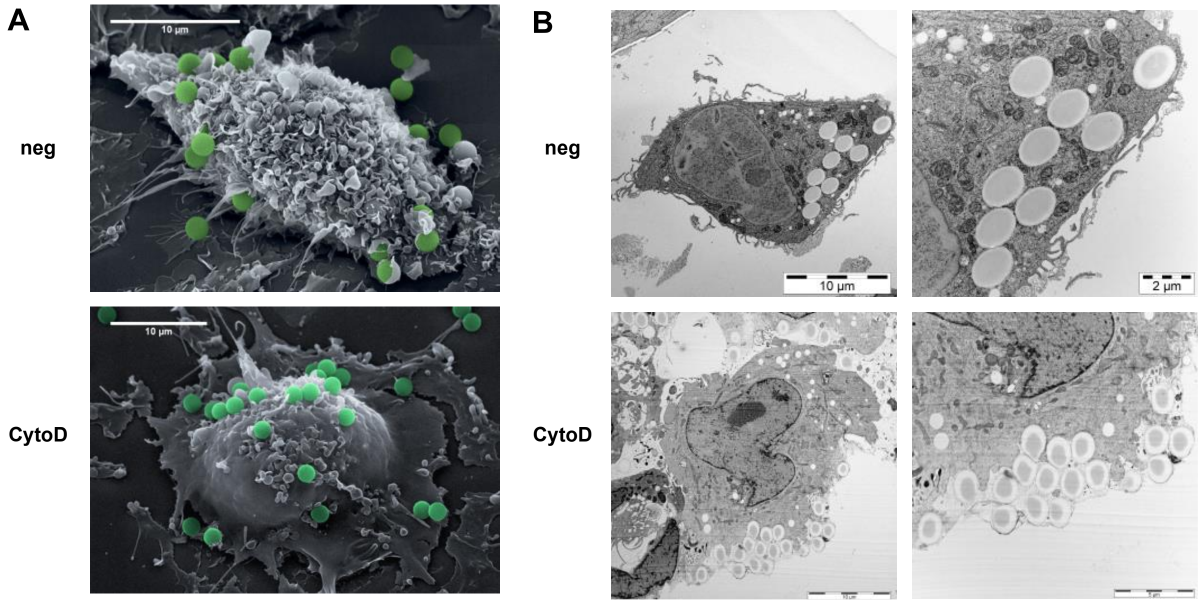

<u>Supplementary Figure S1:</u> Electron microscopy to confirm reduced levels of intracellular latex beads in PMA-activated THP-1 MΦ treated with cytochalasin D. (A) SEM and (B) TEM images of untreated (neg) or cytochalasin D-treated PMA-activated (CytoD) THP-1 MΦ.

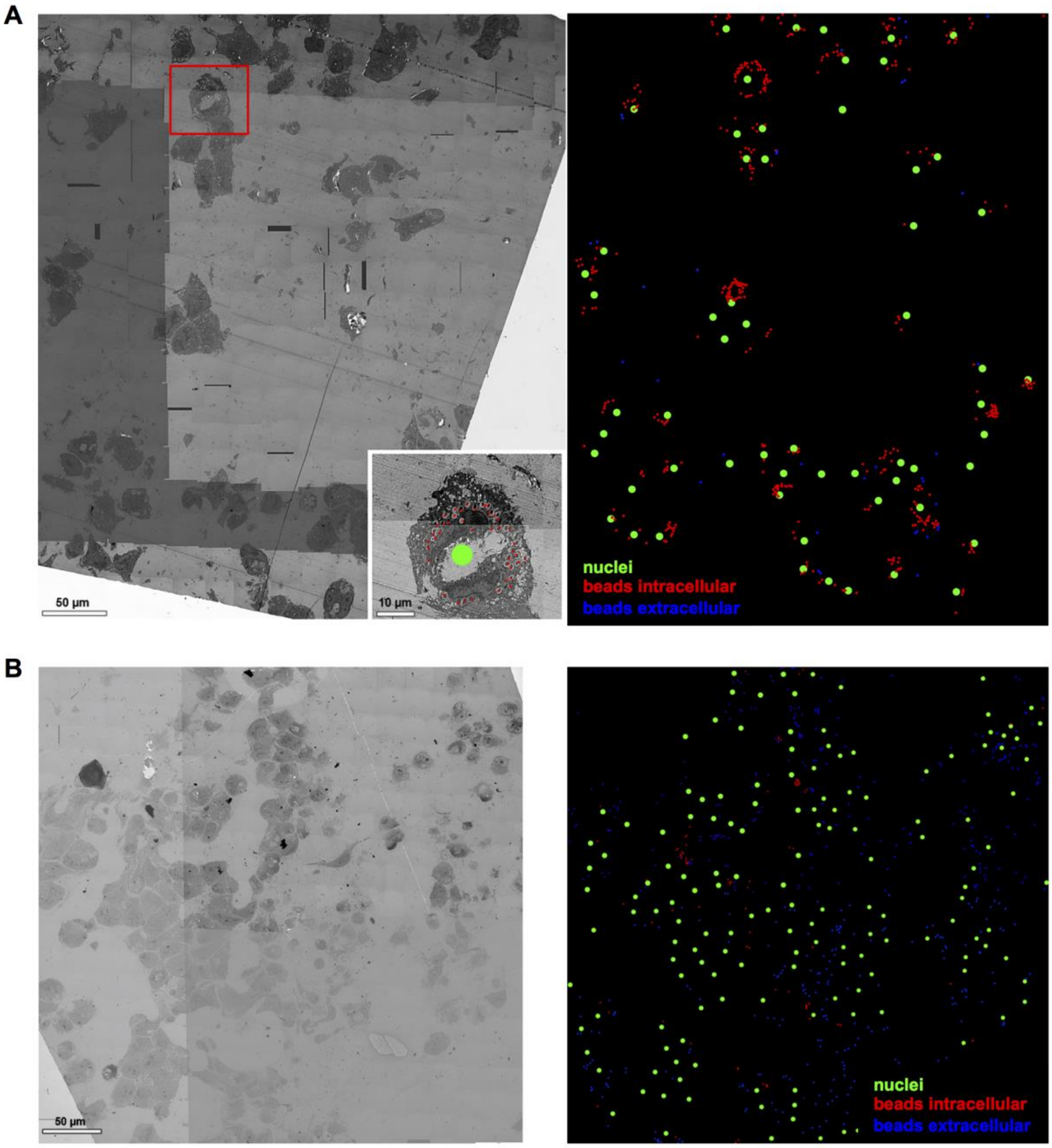

<u>Supplementary Figure S2:</u> Electron microscopy of PMA-activated THP-1 cells phagocytosing latex beads. TEM panorama images of untreated (A) or cytochalasin D-treated, PMA-activated THP-1 Mq (B). Individual, high resolution images were aquired on a Zeiss 912 transmission electron with 14 nm per pixel and collated together to a panoramic overview across the entire specimen (left panels). These images were imported into Adobe Photoshop CC 2017 and processed by labeling of nuclei with green dots, intracellular beads by red dots and extracellular beads by blue dots as shown in the inset in (A). Subsequently, the image

background was replaced by a uniform black background (right panels), and green, red, and blue dots were automatically to provide the numbers of cells, intra- and extracellular beads.

A

	neg	CytoD
Total cells	60	155
Intracellular beads	394	84
Extracellular beads	37	482
% intracellular beads	91.4 %	14.8 %
Intracellular beads/cell	6.6	0.5
Extracellular beads/cell	0.6	3.1

B

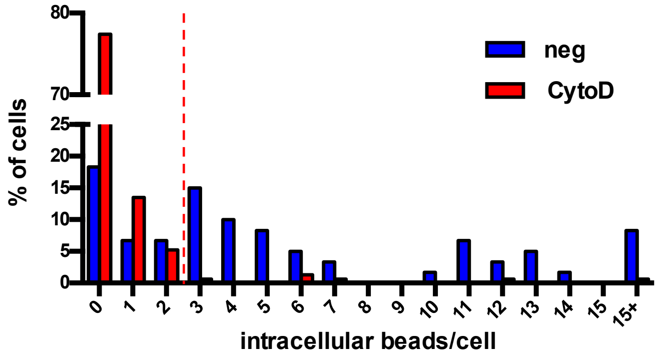

<u>Supplementary Figure S3:</u> **Quantitative image analysis on intra- and extracellular beads counted in cells of panorama TEM images shown in Supplementary Figure S2).** Values in (A) are cumulative numbers of cells and beads counted in all cells analyzed whereas values in (B) are percentage of cells with the indicated number of intracellular beads.

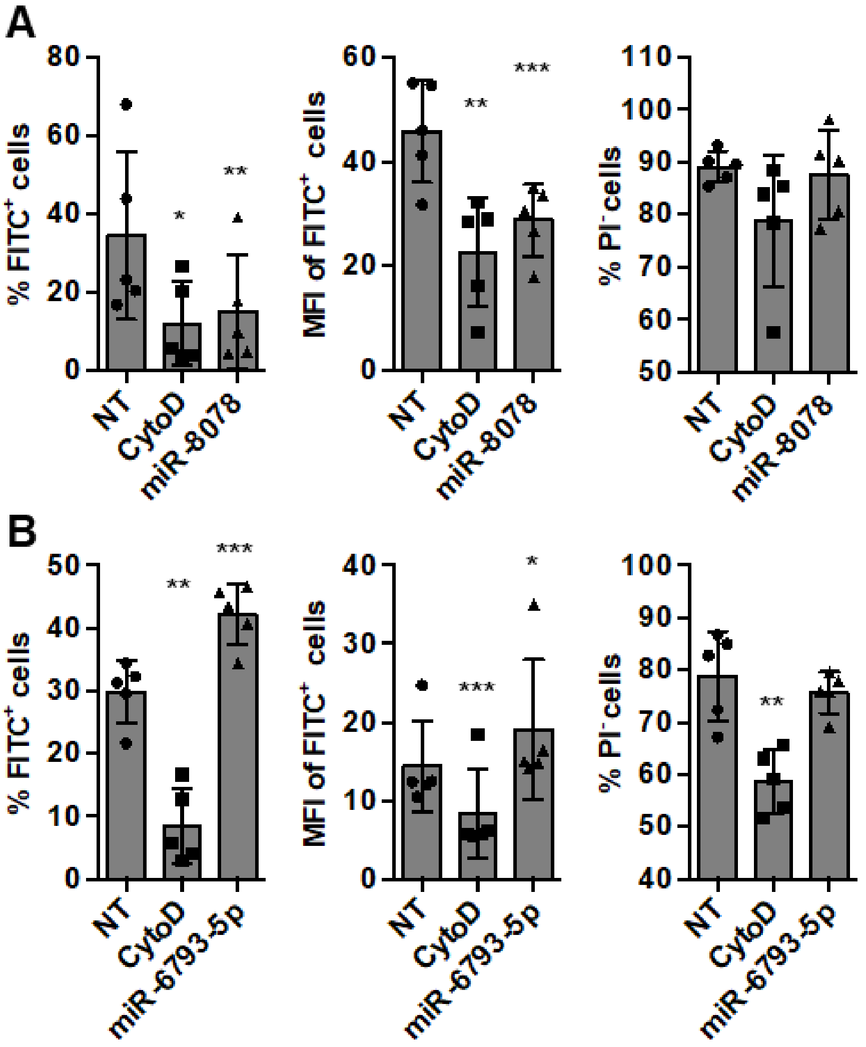

<u>Supplementary Figure S4:</u> Validation of the results obtained in the screens for miR-8078 and miR-6793-5p. Phagocytosis of FITC-labeled, opsonized latex beads by PMA-activated THP-1 MΦ treated with an NT siRNA (NT), cytochalasin D (CytoD) or mir-8078 (A) or miR-6793-5p (B) as percentage of phagocytic cells (% FITC^{+} cells, left), phagocytic activity (MFI of FITC^{+} cells, middle) and viability (% PI^{-} cells) across n = 5 independent experiments. Statistical analysis was performed using repeated measures, one-way ANOVA with Geisser-Greenhouse correction. Dunnett's post analysis was used to calculate *p*-values adjusted for multiple comparisons with the NT set as control condition (*: $p < 0.05$; **: $p < 0.01$; ***: $p < 0.001$)

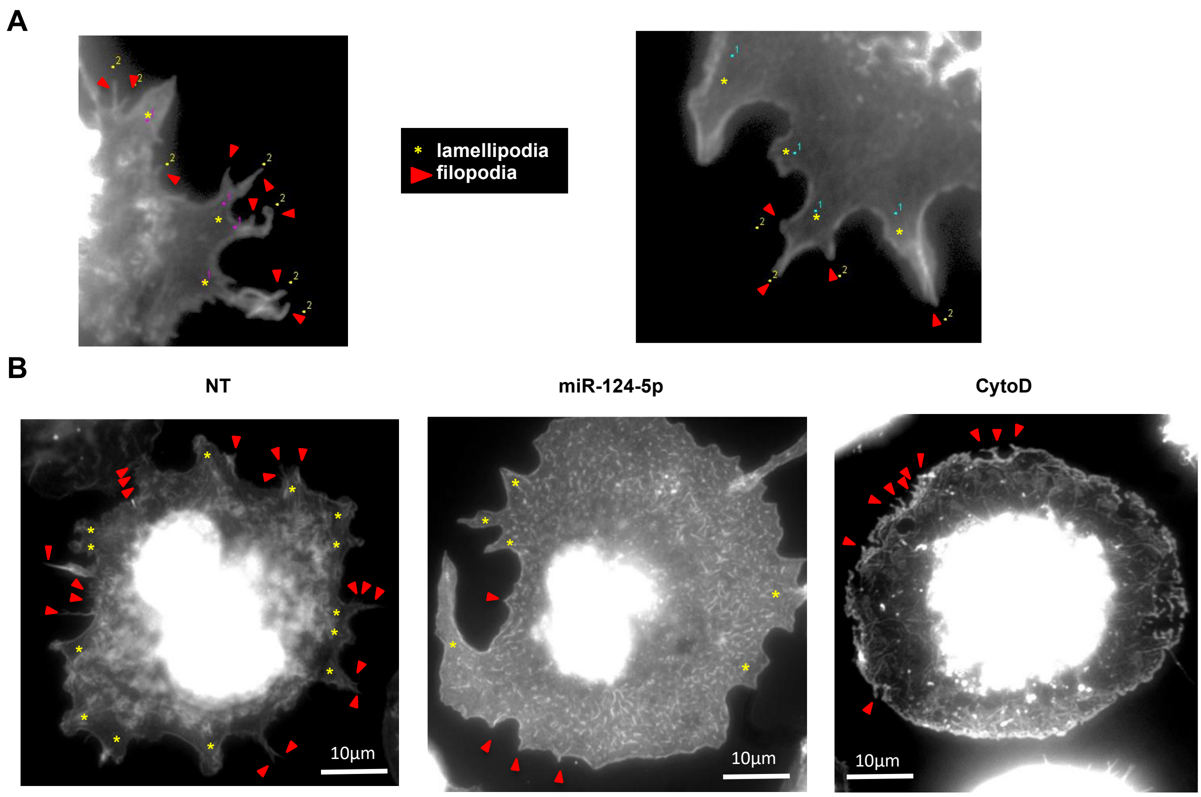

<u>Supplementary Figure S5:</u> Fluorescence microscopic analysis of the effect of miR-124-5p on formation of filopodia and lamellipodia. Fluorescence microscopy of PMA-activated THP-1 MΦ following the indicated treatments. Actin cytoskeleton was stained with Alexa Fluor 488-phalloidin and nuclei with Hoechst 33342. Images were aquired with a Zeiss AxioObserver Z1 using a 100× objective (scale bars: 10 µm) and fluorescence intensity of phalloidin-stained actin is shown. (A) Digital magnification on a parts of two NT-treated control cells to illustarte how lamellipodia (asterisks) and filopodia (red triangles) were manually labeled for subsequent image analysis. (B) Representative cells for each condition. Labeled lamellipodia and filopodia were automatically counted using the ImageJ software. Quantitative analysis of 47-57 individual cells across n = 5 independent experiments are shown in Figure 5B.

Supplementary Tables

Supplementary Table S1: Predicted binding sites of miR-124-5p in potential target gene mRNAs cloned into a pmirGLO® Dual Luciferase miRNA target expression vector.

Gene	Target sequence	Location[1]	ΔG [°C][2]	Database
ARPC1A	CAACAUGUCUGCCAUGGAACGCU	1009-1031 (CDS)	-22.4	RNA22 v2.0
ARPC1B	AACAAGUUUGCUGUGGGCAGCG	634-655 (CDS)	-28.1	RNA22 v2.0
ARPC2	AAGCGGCUGGCAACUGAAGGCUGGAACACUUGCUACUG	993-1025 (3'-UTR)	-23.5	miRmap
ARPC3	UGUGAAGAGACAGUUCAUGCGUAA	583-604 (CDS)	-13.8	STarMir
ARPC4	GAAAAGGUUCUGAUUGAGGGCU	237-258 (CDS)	-19.4	RNA22 v2.0
ACTR2	GGUGAUGAGGCAAGUGAAUUACG	260-283 (CDS)	-16.5	miRWalk2.0

[1] Position of predicted binding motifs with nucleotides numbers and its location in the coding sequences (CDS) or 3' untranslated region (3'UTR) of target mRNA.
[2] Binding energy of the interaction with miR-124-5p and the respective target sequence predicted by the STarMir algorithm.

Supplementary Table S2: Oligonucleotide primers used for qRT-PCR quantification of target gene expression levels.

oligonucleotide	Sequence (5' → 3′)	Reference[1]
**qRT-PCR primers**		
ARPC1A_fwd<br>ARPC1A_rev	ATTGCCCTCAGTCCCAATAATCA<br>CAAGTGACAATGCGGTCGC	300360514c1
ARPC1B_fwd<br>ARPC1B_rev	CAAGGACCGCACCCAGATT<br>TGCCGCAGGTCACAATACG	325197176c1
ARPC2_fwd<br>ARPC2_rev	GCAGATTTCGATGGGGTCCTC<br>ACTCCCGTACACCCTCTTTAAT	23238210c2
ARPC3_fwd<br>ARPC3_rev	GTGCAATTCCAAAAGCCAAGG<br>GGCTCTCATCACTTCATCTTCC	23397667c1
ARPC4_fwd<br>ARPC4_rev	AACGACACAACAAGCCGGAA<br>GGCACAAAATCTTCTCGATCTCA	5031595a3
ARPC5_fwd<br>ARPC5_rev	TGGTGTGGATCTCCTAATGAAGT<br>CACGAACAATGGACCCTACTC	23238212c1
ARPC5L_fwd<br>ARPC5L_rev	ATCGACGAATTTGACGAGAACA<br>GAATGCCCGAAGCATGTCC	13569955c1
ACTR2_fwd<br>ACTR2_rev	GGCAGTTCTGACTTTGTACGC<br>CCAGTCTCCTGGTAAGATGAGG	205361120c2
ACTR3_fwd<br>ACTR3_rev	GGAGAACGGACGTTGACCG<br>TCCTGCGATTGGAATGTGTTT	34452698c1
GAPDH_fwd<br>GAPDH_rev	GAGTCAACGGATTTGGTCGT<br>TTGATTTTGGAGGGATCTCG	NCBI Primer BLAST

1: numbers indicate IDs in PrimerBank (https://pga.mgh.harvard.edu/primerbank/).

Supplementary Table S3: Oligonucleotide primers used cloning of predicted wildtype or mutated miR-124-5p binding sites for dual luciferase assays to probe miRNA-mRNA interactions.

oligonucleotide	Sequence (5' → 3′)	Reference
Right_universal_rev	CTTTCCGCCTCAGAAGGTAC	this study
Left_universal_fwd	CGTCCGGCGTAGAGGATCGA	this study
ARPC1A_WT_right_fwd	CAACATGTCTGCCATGGAACGCTCCTCGAGTCTAGAGTCGACCTG	this study
ARPC1A_WT_left_rev	AGCGTTCCATGGCAGACATGTTGCTAGCGAGCTCGTTTAAACA	this study
ARPC1B_WT_right_fwd	AACAAGTTTGCTGTGGGCAGCGCCTCGAGTCTAGAGTCGACCTG	this study
ARPC1B_WT_left_rev	CGCTGCCCACAGCAAACTTGTTCTAGCGAGCTCGTTTAAACA	this study
ARPC2_WT_right_fwd	GGCAACTGAAGGCTGGAACACTCCTCGAGTCTAGAGTCGACCTG	this study
ARPC2_WT_left_rev	AGTGTTCCAGCCTTCAGTTGCCCTAGCGAGCTCGTTTAAACA	this study
ARPC3_WT_right_fwd	GAAGAGACAGTTCATGAACAAGACCTCGAGTCTAGAGTCGACCTG	this study
ARPC3_WT_left_rev	TCTTGTTCATGAACTGTCTCTTCCTAGCGAGCTCGTTTAAACA	this study
ARPC3_MUT_right_fwd	GAAGAGACAGTTCATGCGTAAGACCTCGAGTCTAGAGTCGACCTG	this study
ARPC3_MUT_left_rev	TCTTACGCATGAACTGTCTCTTCCTAGCGAGCTCGTTTAAACA	this study
ARPC4_WT_right_fwd	GAAAAGGTTCTGATTGAGGGCTCCTCGAGTCTAGAGTCGACCTG	this study
ARPC4_WT_left_rev	AGCCCTCAATCAGAACCTTTTCCTAGCGAGCTCGTTTAAACA	this study
ARPC4_MUT_right_fwd	GAAAAGGTTCTGATAATTAAATCCTCGAGTCTAGAGTCGACCTG	this study
ARPC4_MUT_left_rev	ATTTAATTATCAGAACCTTTTC CTAGCGAGCTCGTTTAAACA	this study
ACTR2_WT_right_fwd	GGTGATGAGGCAAGTGAATTACGCCTCGAGTCTAGAGTCGACCTG	this study
ACTR2_WT_left_rev	CGTAATTCACTTGCCTCATCACCCTAGCGAGCTCGTTTAAACA	this study
